# How Do Preservice Teachers Make Sense of Educational Inequalities? Exploring Critical Consciousness Through Mixed Methods

**DOI:** 10.1002/jcop.70070

**Published:** 2025-12-02

**Authors:** Yağmur Güleç, Miriam Schwarzenthal, Tuğçe Aral

**Affiliations:** ^1^ School of Education University of Wuppertal Wuppertal North Rhine‐Westphalia Germany; ^2^ Department of Inclusive Education University of Potsdam Potsdam Brandenburg Germany

**Keywords:** critical actions, critical consciousness, educational inequalities, mixed‐methods, preservice teachers, self‐efficacy, structural attributions

## Abstract

This study adopted a mixed‐methods approach to explore the critical consciousness (CC) of German preservice teachers regarding educational inequalities related to socioeconomic status (SES) and family migration history. We examined how CC manifests in preservice teachers, how the CC subcomponents relate to each other, and what factors predict CC. Preservice teachers (*N* = 93, *M*_age_ = 24.29 years, SD_age_ = 5.77) responded to open‐ended questions capturing their CC and completed rating‐scale measures of potential predictors (i.e., discrimination, taken classes on diversity and equity, social dominance orientation, subjective SES). Qualitative analysis revealed preservice teachers attributed educational inequalities to individual, structural, and parental capital factors. Proposed actions to reduce educational inequalities included equality and inclusion, individual support, critical action, and multicultural education. Quantitative analysis indicated discrimination experiences predicted critical action intentions. Understanding preservice teachers’ CC can guide targeted interventions and teacher training programs that equip them to effectively address educational inequalities.

Educational systems are not fair. Differential distribution of financial and educational resources, discrimination grounded in systems of oppression such as sexism, racism, and classism, and educational policies (e.g., assimilative, exclusive) contribute to the achievement gap between students with low versus high socioeconomic status (SES) and with or without a family history of migration (OECD 2023). Given the systemic barriers preventing students from succeeding, the term “opportunity gap” more accurately reflects educational inequalities through an equity lens rather than blaming the students themselves (Gorski and Pothini 2018). Germany exemplifies opportunity gaps, with students from lower SES backgrounds or with a family history of migration being less likely to attend the highest school track (i.e., Gymnasium) and pursue higher education (Autor:innengruppe Bildungsberichterstattung Ed. 2024; Lewalter et al. 2023).

Whereas previous literature has emphasized ‘fixing’ the students, research increasingly highlights the importance of teachers’ psychology (e.g., beliefs, judgments, assessments, perceptions) for student's success (Turetsky et al. 2021). To reduce educational inequalities, teachers should critically reflect on these disparities (critical reflection), take action to address them (critical action), and feel both prepared and self‐efficacious to reduce inequalities (self‐efficacy) (Gay and Kirkland 2003). This paper aims to explore how critical reflection, critical action, and self‐efficacy manifest among preservice teachers in Germany and how they are interrelated, as well as to identify their antecedents. Understanding these components is essential as teachers’ critical reflection, action, and self‐efficacy are key factors that shape classroom dynamics and influence student outcomes (e.g., achievement, self‐esteem), especially for marginalized students (Seider and Graves 2020). Teachers account for up to 30% of the variance in student performance through instructional quality and classroom climate (Hattie 2003). As future decision‐makers, preservice teachers will interpret achievement, make tracking recommendations, and influence equity through their awareness of social positioning and expectations.

## 1 Critical Consciousness Theory

To examine teachers’ critical reflection, critical actions, and self‐efficacy in the context of educational inequalities within one comprehensive framework, this paper draws on the framework of critical consciousness (CC) (Freire 1970/2018). Through developing CC, people reflect on systemic power disparities, which can subsequently inspire action, enabling oppressed individuals to achieve liberation. Rather than a passive awareness, CC emphasizes the development of an active, engaged stance toward societal transformation.

CC consists of three sub‐components: critical reflection, critical action, and self‐efficacy (sometimes also called critical motivation) (Watts et al. 2011). Critical reflection is typically defined as reflecting on power disparities in society that marginalize groups of people (Diemer et al. 2016; Heberle et al. 2020; Watts et al. 2011), whereas critical action comprises behaviors to redress oppression and promote social justice (Mathews et al. 2023). Lastly, political efficacy refers to the belief in the capacity to dismantle social inequities, which may serve as a link between critical reflection and action (Watts et al. 2011). Together, these components form a dynamic process through which individuals become aware of structural injustices and feel empowered to address them.

Originally conceptualized as a process of empowerment for oppressed populations, CC has since been applied more broadly to assess how individuals, including those in more privileged positions, can develop a deeper awareness of social injustices and contribute to equity‐oriented change. Those with greater power and resources can collaborate with marginalized communities to achieve collective liberation (Thomas et al. 2014).

## 2 Teacher's Critical Consciousness in the Context of Educational Inequalities

In Germany, preservice teachers are often middle‐class, ethnic‐majority women (Middendorff et al. 2017). It is crucial for them to be aware of how their privileged identities may entail experiences that differ from those of their marginalized students and to recognize how power imbalances shape their students’ academic performance, which may increase their likelihood of engaging in actions to reduce educational inequalities (Chubbuck 2010). Nevertheless, people from privileged backgrounds often reproduce what Bonilla‐Silva (2003/2021) terms color‐blind racism, i.e., denying structural racism by claiming “I don't see race” or that racism belongs only to the past. In Germany, this perspective is common in public discourse and among teachers, where racism is often seen as limited to the Nazi era rather than a present‐day issue (Alexopoulou 2021; Burhoff 2021). The CC framework offers a valuable lens for examining how preservice teachers in this context recognize and respond to educational inequalities.

## 3 Teachers’ Attributions of Social Inequalities

Critical reflection is often equated with attributing inequalities to structural factors (Godfrey and Burson 2018; Godfrey and Wolf 2016; Watts et al. 2011). In the context of educational inequalities, students’ abilities and efforts represent typical individual attributions, whereas the educational system and discrimination represent typical external or structural attributions (Froehlich et al. 2016). Therefore, in this study, we understand critical reflection as attributing the opportunity gaps between students with high versus low SES, and with or without a family history of migration to structural factors such as the educational system and discrimination. Many teachers already make structural attributions of educational inequalities. Experimental studies on attributions of the opportunity gap in Germany showed that preservice teachers tend to attribute the lower success of students with Turkish roots to external factors (e.g., educational system, stereotyping) more than to internal factors (e.g., intellectual ability, motivation) (Glock and Schuchart 2020).

Even though quantitative research on critical reflection often distinguishes between individual and structural attributions, qualitative studies reveal more nuanced forms within and sometimes beyond this dichotomy. In the U.S., for example, preservice teachers have attributed racial disparities in education to structural factors, such as institutional and interpersonal racism, as well as to individual‐level explanations like victim blaming and deficit thinking (Tanase and Gorski 2025). Behrmann (2021) found that in Germany, using interviews, teachers not only attributed academic performance to individual or teacher‐related factors but also to parental factors (e.g., parental involvement). According to Bourdieu (1986), parental influence can be understood through the concepts of cultural, social, and economic capital**—**resources such as knowledge, skills, and values (cultural capital); social networks or connections (social capital); and material assets or financial means (economic capital) that families provide, all of which can significantly affect students’ educational outcomes and opportunities.

In addition, some German teachers locate the causes of students’ educational behaviors and achievement in their cultural backgrounds—for example, by attributing Turkish‐heritage students’ behaviors to assumed cultural traits (Moffitt et al. 2019). Though framed as group‐level attributions, such views often function similarly to individual attributions by essentializing cultural groups and implying fixed, internal deficits. These attributional patterns, sometimes referred to as “cultural racism” (Fereidooni and El 2017), shift responsibility to marginalized individuals, rather than addressing structural inequalities. Hence, in previous research, explanations invoking a “culture of poverty” or “how people were raised” were grouped with individual attributions, as they focus on presumed inherent deficiencies of the individual or their upbringing, rather than systemic factors (Hershberg and Johnson 2019).

In addition to examining teacher attributions beyond the individual versus structural binary, it is important to consider the number of attributions they provide, as this may reflect their knowledge level or the complexity of their understanding (Flanagan et al. 2014). To capture the full range of preservice teachers’ explanations for educational inequalities, this study used a qualitative approach to extract their open‐ended responses, which were then quantified to examine the variety and frequency of the explanations provided.

It remains unclear whether different types of inequality, such as economic or racial, elicit distinct attribution patterns in preservice teachers. Although CC has been reliably measured across domains like race and class (Diemer et al. 2017), individuals may reflect more critically on some issues than others (e.g., racism vs. sexism; Diemer et al. 2015; Godfrey and Burson 2018). This study, therefore, examines whether preservice teachers’ structural attributions apply similarly to opportunity gaps related to SES and a family history of migration background.

## 4 Teachers’ Actions to Address Social Inequalities

While critical reflection helps preservice teachers recognize structural issues, meaningful change requires concrete action. Although empirical examples are limited, conceptual frameworks such as critical pedagogy (Freire 1970/2018), critical literacy (Han 2013), culturally responsive teaching (Gay 2018), multicultural education (Banks and Banks 2019), equity literacy (Gorski and Pothini 2018), and culturally relevant pedagogy (Ladson‐Billings 1995) offer guidance. The latter, for instance, highlights students’ sociopolitical consciousness and positions education as a tool for social transformation. For preservice teachers, this entails reflecting on power and privilege and helping students critically engage with inequality (Ladson‐Billings and Henry 1990; Freire 1970/2018).

Building on these concepts, research on German teachers’ work with culturally diverse students identifies two main approaches: “equality and inclusion” (focusing on similarities and cooperation) and “cultural pluralism” (emphasizing cultural differences and CC socialization) (Schachner 2019). In German classrooms, “equality and inclusion” dominates, likely due to the assimilative school culture (Civitillo et al. 2017; Schwarzenthal et al. 2022). Therefore, we expect German preservice teachers to prioritize action intentions related to equality and inclusion over those emphasizing cultural pluralism (i.e., multicultural education or CC socialization).

Among classroom actions to address opportunity gaps, CC socialization and sociopolitical consciousness best fit definitions of critical action (Mathews et al. 2023), as fostering student reflection on social inequality can help change inequitable structures. CC socialization involves the processes through which individuals develop CC components. For example, a school climate promoting open dialog, challenging stereotypes, and supporting student initiatives fosters awareness and agency (Schwarzenthal et al. 2022). Beyond the classroom, preservice teachers can also engage in critical actions such as participating in demonstrations and campaigns for systemic educational reform (Cho and Choi 2016). Thus, we define teachers’ critical actions as efforts to reduce educational inequalities both by fostering CC socialization in schools and advocating for policy and structural changes. This study employs a qualitative approach to explore the range of actions preservice teachers propose, including equality and inclusion, multiculturalism, and critical action.

## 5 Teachers’ Self‐Efficacy to Address Social Inequalities

Beyond the actions teachers take, their self‐efficacy, i.e., the belief in their ability to influence student outcomes, also plays an important role in educational settings and, according to CC literature, may strengthen the relation between critical reflection and action (Watts et al. 2011). High teacher self‐efficacy is linked to positive attitudes toward inclusion, higher tracking recommendations (Geven et al. 2018), and the adoption of new instructional practices (Zee and Koomen 2016). However, self‐efficacy varies across teaching domains, such as learning difficulties and classroom management (Tschannen‐Moran and Hoy 2001), and across student groups (e.g., ethnic minority vs. majority students; Geerlings et al. 2018). Addressing educational inequalities requires teacher self‐efficacy not just for individual student performance but for closing opportunity gaps and tackling structural barriers. This study thus focuses on teachers’ perceived capacity to address such gaps. Yet, many educators feel unprepared for cultural diversity (Civitillo 2019), and research on self‐efficacy in this area remains scarce.

## 6 Interrelations between the Subcomponents of CC

Freire assumed that gaining more knowledge and awareness regarding societal issues may lead to more critical action and vice versa (Freire 1970/2018). However, empirical findings are still unclear, as critical reflection and action are correlated in some (Clark and Seider 2020; Diemer and Rapa 2016), but not in all studies (Diemer et al. 2017). Similarly, in an educational context, Chubbuck (2010) framework suggests that preservice teachers’ action orientation—whether fair or deficit‐oriented—depends on whether they attribute gaps to individual or structural factors. While individual attributions alone do not necessarily lead to deficit‐oriented actions, a combination of individual and structural attributions is crucial to prevent deficit‐oriented approaches and support equitable solutions. As one of only a few studies examining preservice teachers’ attributions and actions, Kehl et al. (2024) conducted interviews with five teachers in Germany regarding their beliefs concerning racially minoritized students. However, no link was observed between beliefs (individual, i.e., deficit and differentiation thinking vs. structural, i.e., critical reflection) and culturally responsive teaching practices. More studies are needed to shed light on the relation between preservice teachers’ critical reflection and action.

However, many studies observed a positive link between a strong sense of political self‐efficacy and being engaged in critical action among diverse groups of adolescents (Seider and Graves 2020; Christens and Dolan 2011; Corcoran et al. 2015; Hope and Jagers 2014; Diemer and Rapa 2016). This relationship can be explained by the social changes that emerge following critical actions (Seider and Graves 2020): When adolescents observe that their actions lead to tangible outcomes, they are more likely to be motivated to participate in future actions. Conversely, those with higher self‐efficacy may already feel more capable and thus be more inclined to take action. Nevertheless, to date, we know little about the links between preservice teachers’ efficacy in reducing educational inequalities and their critical action. Similarly, little is known about the relation between preservice teachers’ critical reflection and their self‐efficacy to reduce educational inequalities.

### 6.1 Antecedents of CC

To support preservice teachers’ CC development, it is crucial to understand its potential antecedents. CC theory (Freire 1970/2018) conceptualizes CC as a developmental process shaped by individual experiences, social contexts, and access to reflection and action opportunities. Through engaging with their environments (e.g., school, family, or community), people learn to “read the word and the world” and recognize injustice. Watts et al. (2011) highlight that CC develops through interaction with ecological systems, including social structures and power relations. Access to sustained opportunities for dialog and equity‐focused education supports this process. Thus, CC theory provides a framework for examining predictors such as discrimination experiences, exposure to equity content, and sociopolitical positioning (e.g., SES, SDO).

#### 6.1.1 Discrimination

Rooted in CC theory, unfair treatments like discrimination can be pivotal in developing awareness of power disparities in society, which, in turn, may motivate individuals to take action against social injustices (Freire 1970/2018; Anyiwo et al. 2018; Kelly 2018). Studies with marginalized youth showed that discrimination is positively associated with critical reflection (Gale et al. 2023; Tyler et al. 2020) and critical action (Gale et al. 2023; Hope et al. 2018; Kehl et al. forthcoming; Tyler et al. 2020; Schwarzenthal et al. 2024). However, associations between discrimination and critical motivation varied, with one study finding no correlation among Black youth (Hope and Jagers 2014), whereas others did find such a relationship in both CC and civic engagement studies (Hope et al. 2018; Rubin 2007). Further research is needed to clarify the relationship between discrimination and critical motivation.

#### 6.1.2 Taken Classes on Diversity and Equity‐Related Topics

Prior education about societal structures may shape critical understanding regarding power disparities (Heberle et al. 2020). In Freire's dialogic pedagogy approach, the teacher's absolute authority over students is dissolved, transforming education from a one‐way transmission of knowledge into a shared process of learning (Freire 1970/2018). This collaborative process fosters critical reflection, helping learners understand systemic inequalities and power disparities in society.

Similar to the dialogic approach, when CC socialization was implemented in schools, which included discussions and training on diversity and equity‐related topics, this was accompanied by improvements in students’ critical reflection and motivation (Schwarzenthal et al. 2022; Seider et al. 2016). Additionally, Flanagan et al. (2014) found that adolescents with classmates who discuss societal issues with their families offered more explanations for economic inequalities. However, evidence linking CC climates to critical action is mixed (Bañales et al. 2021; Schwarzenthal et al. 2022; Schwarzenthal et al. 2024).

Importantly, while much research has focused on adolescents, recent systematic reviews highlight that initial teacher education programs vary widely in preparing preservice teachers to address social justice in schools (Keles and Munthe 2025). Some studies indicated that diversity and social justice courses may enhance preservice teachers’ critical awareness of systemic inequities as well as their own privileges (e.g., Aronson and Meyers 2022), particularly when such courses are sustained over a longer period (Hazzard 2021). Nevertheless, more research is needed to understand how these educational experiences translate into critical reflection and action among preservice teachers, particularly in different sociocultural contexts.

#### 6.1.3 Social Dominance Orientation (SDO)

Contrary to CC, SDO refers to a preference to maintain the status quo and power hierarchies in society (Pratto et al. 1994). While Diemer et al. (2006) have used inverted SDO scores as a proxy for critical reflection, we followed more recent work that conceptualized SDO as a broader socio‐political orientation that may shape, but is distinct from, the components of CC (Puckett et al. 2020). SDO showed a positive relationship with the absence and precritical stage of CC as assessed with a Guttman scale (Thomas et al. 2014). People with high SDO showed lower critical reflection and action (Puckett et al. 2020), lower rates of volunteering and collective action (Oosterhoff et al. 2017), and reduced critical motivation (Carvalho et al. 2021). Thus, higher SDO has been quite consistently related to lower CC, even though studies examining this relation among preservice teachers are still lacking.

#### 6.1.4 Subjective SES

Unlike objective SES, which is typically based on parental education, occupation, or income, subjective SES refers to individuals’ perceptions of their own socioeconomic standing relative to others, often measured using self‐placement on a social ladder (e.g., Adler et al. 2000). This perception can shape worldviews and responses to inequality independently of actual economic resources (Aydin and Vera 2020). Although most prior studies examining the relationship between SES and attributions of inequalities have relied on objective indicators (e.g., Flanagan et al. 2003, 2014; Kornbluh et al. 2019), our study employs subjective SES, which may better capture psychological processes and class experiences related to identity, perceived privilege, and social comparison, all of which are potentially relevant for understanding the development of CC (Aydin 2016).

However, whether low SES fosters or hinders CC remains an open question. According to CC theory, structural disadvantage may enhance individuals’ awareness of inequality and motivate action to address it (Freire 1970/2018). In contrast, system justification theory posits that people with low SES may be more inclined to legitimize societal hierarchies, reducing critical reflection to maintain psychological stability and hope for mobility (Jost and Banaji 1994; Godfrey and Wolf 2016). Empirical findings align with system justification: low SES adolescents more often attributed inequality to individual causes compared to high SES youth (Flanagan et al. 2003, 2014; Kornbluh et al. 2019), and showed lower levels of critical motivation (Choi et al. 2023). Higher SES was also associated with greater critical reflection (Bañales et al. 2021) and interpersonal critical action (Schwarzenthal et al. 2024).

From a civic engagement perspective—closely related to critical action—low SES has often been linked to lower political and civic participation due to limited access to resources and education to take action (Campbell 2009; Lechner et al. 2018; Hope and Jagers 2014). However, more recent studies among low SES young adults have found higher civic engagement compared to high SES young adults (Godfrey and Cherng 2016; Sánchez‐García et al. 2024; Wray‐Lake et al. 2016), suggesting that marginalization can also foster agency for a fairer society.

One reason for these mixed findings may be the inconsistent operationalization of both SES and CC subcomponents. To our knowledge, no existing research has directly examined the link between CC and subjective SES, limiting our understanding of how perceived class status is related to CC development.

### 6.2 Current Study

The present study adopted a mixed‐methods approach to investigate (1) how CC manifests in preservice teachers, (2) how CC components are related, and (3) what factors predict CC. To better understand these dynamics, it is important to consider the specific socio‐political and educational context in which preservice teachers are being trained.

#### 6.2.1 The German Educational Context

Germany's school population has become increasingly diverse, with 29% of pupils having a migration background, compared to just 11% of teachers (Statistisches Bundesamt 2024), creating a demographic gap that influences classroom dynamics (Glock and Schuchart 2020). Germany's early tracked school system further reinforces inequalities: children are placed into vocational (i.e., *Hauptschule, Realschule*) or academic tracks (i.e., *Gymnasium*) after primary school, with placement closely tied to parental SES and migration history: While 78% of children from high‐SES families receive a Gymnasium recommendation, only about one‐third of low‐SES children receive it. Likewise, 44% of adolescents without a migration background attend Gymnasium, versus just 30% with a migration background (Autor:innengruppe Bildungsberichterstattung Ed. 2024; Lewalter et al. 2023).

In Germany, educational privilege is shaped by intersecting factors such as social origin, gender, and migration history. At the same time, public discourse typically avoids racial categories and instead uses terms like “migration background” (El‐Mafaalani 2022; Elrick and Farah Schwartzman 2015). Within this context, preservice teachers are often middle‐class, ethnic majority women, making them a relatively privileged group (Middendorff et al. 2017).

Compared to the US, with its stronger emphasis on economic liberalization and weaker social protections, Germany offers a more expansive welfare state and free access to education (Garritzmann 2016). Instead, its policy approach has focused more on formal equality and inclusion—strategies shaped in part by the country's post–World War II sociopolitical context (Civitillo et al. 2017).

#### 6.2.2 Research Questions and Hypotheses

**Figure 1:**
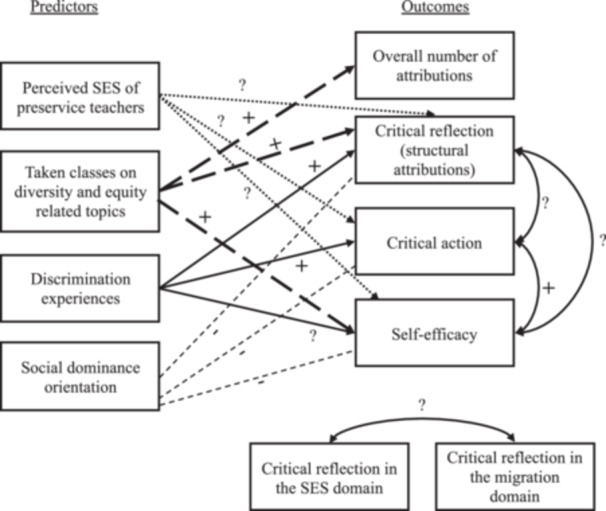
*Illustrations of potential links between predictors and outcome variables. Note*. Different arrow styles were used for each predictor to enhance clarity and prevent confusion. Question marks indicate exploratory relationships.

We addressed the following research questions (Figure 1):

RQ1 (Qualitative) How does CC manifest in the attributions and action intentions of preservice teachers regarding educational inequalities? Based on prior literature, we expected participants to attribute inequalities to individual (including cultural), parental, and structural factors, and to suggest action strategies such as equality and inclusion, multicultural education, and critical action, with equality and inclusion being the most commonly mentioned.

RQ2 (Quantitative) How are the subcomponents of CC related to one another?

Critical action and self‐efficacy to reduce educational inequalities will be positively associated. We further explored (exploratory) the relations between structural attributions (critical reflection) and both critical action and self‐efficacy, given mixed results in prior research. We also explored associations between structural attributions across the SES and migration domains.

RQ3 (Quantitative) What predicts the development of CC in preservice teachers?

Discrimination experiences will be positively associated with structural attributions (critical reflection) and critical action. We also explored associations with self‐efficacy. *H2b:* Having taken courses on equity and diversity‐related topics will be positively associated with structural attributions (critical reflection), self‐efficacy, and the overall number of attributions (quantity). *H2c:* SDO will be negatively associated with all CC subcomponents. Finally, due to inconsistent findings in previous literature, we explored the relation between subjective SES and CC.

#### 6.2.3 Contributions and Significance of the Study

Our study makes several contributions to the literature. First, while most CC research has focused on adolescents (Heberle et al. 2020), we examine preservice teachers (i.e., young adults) who possess greater political agency and play a central role in shaping equitable education (Freire 1970/2018; Bañales et al. 2020). Second, we apply the CC framework to study teachers’ attributions, actions, and efficacy within a unified approach, integrating previously separate research strands. Third, we address a gap in the literature by examining CC in the underexplored context of Germany, thereby extending the applicability of the CC framework beyond predominantly U.S.‐based research. Fourth, our mixed‐methods design allows for both nuanced insights and broader generalizability regarding preservice teachers’ understanding of educational inequality (van Griensven et al. 2014).

## 7 Materials and Methods

### 7.1 Sample and Procedure

Data were collected through an online questionnaire using the software Unipark from 93 preservice teachers (*M*_age_ = 24.29 years, SD_*age*_ = 5.77, age range = 18–49) at the beginning and end of summer semester 2021 and winter semester 2021/22 at one university in a medium‐sized city in Eastern Germany. A total of 15 participants reported a migration background, defined as having at least one parent born abroad. The sample comprised 80 women, 11 men, one diverse participant, and one participant who did not indicate gender. They were enrolled either in a bachelor's (*n* = 51) or master's program (*n* = 42). Regarding teaching tracks, 16 were preparing for special‐needs education, 40 for primary education, and 7 for secondary education; 30 did not specify. Subjective SES, assessed on a 10‐point ladder (1 = lowest, 10 = highest), indicated generally privileged backgrounds (*M* = 6.66). Most participants in our sample were without family migration history and with middle‐ to high SES families, consistent with research showing that the majority of preservice teachers in Germany come from educationally privileged environments (Middendorff et al. 2017). (For study materials; https://osf.io/y846a).

The initial aim of the data collection was to evaluate the effects of participating in classes related to social inequity and cultural diversity (i.e., the effect of before vs. after the classes). Due to extensive dropout in measurement point two (*N* = 36), this study only drew on measurement point one (i.e., beginning of the semester). Our post‐hoc power analysis indicated that, with a medium effect size (0.16), four predictors, and two control variables, a sample size of 92 participants would be sufficient for multiple regression analyses. Participants who completed both measurement points had a chance to win one Amazon voucher with a value of 50€. After obtaining ethics approval from the university's ethics committee, the researchers visited the first sessions of two different classes (“Counseling in the School Context” and “Case Studies on Diversity and Social Inequalities”).

### 7.2 Measures

While attributions of educational inequalities and proposed actions to address educational inequalities were measured using open‐ended questions, self‐efficacy and discrimination experiences, taken classes on diversity and equity‐related topics, SDO, and subjective SES of preservice teachers were measured using quantitative scales.

#### 7.2.1 Attributions of Educational Inequalities

We measured attributions of educational gaps using two open‐ended questions: In the SES domain, we asked: “Why do students from higher social classes in Germany perform better at school than students from lower social classes?”, whereas in the migration domain, we asked: “Why do students with a family history of migration perform worse at school than students without?”. We were particularly interested in structural attributions (critical reflection).

#### 7.2.2 Action Intentions

Proposed actions aimed at reducing educational inequalities were assessed with an open question: “What could you do in the future to reduce educational inequalities between students from different groups?” We were particularly interested in actions related to fostering discussions on social inequalities in the classroom or advocating for structural change (critical actions).

#### 7.2.3 Efficacy to Reduce Educational Inequalities

A self‐devised single item measured self‐efficacy to reduce educational inequalities: “How prepared do you feel to address or counteract educational inequalities among students in the school context?” The sliding scale ranged from 0% (*not at all well prepared*) to 100% (*very well prepared*) (standard for recording self‐efficacy). As a single‐item collective efficacy scale showed acceptable convergent reliability with a 20‐item collective efficacy scale in three other studies (Bruton et al. 2016), we also measured self‐efficacy with a single item for reasons of parsimony.

#### 7.2.4 Discrimination

Discrimination was measured using the 9‐item scale by Scheim and Bauer (2019), which captures how often participants experienced unfair treatment based on how they describe themselves or believe others perceive them. This includes a range of social identities, such as ethnicity, gender, religion, income, and more, but the scale does not ask separately about each category. Instead, participants respond to the same set of items (e.g., “You were treated as if you were unfriendly, unhelpful, or rude”) based on their overall perceived identities (ω = 0.82). Response options were 1 (*never*), 2 (*yes, but not in the last 12 months*), 3 (*yes, once or twice in the last 12 months*), 4 (*yes, many times in the last 12 months*).

#### 7.2.5 Taken Classes on Diversity and Equity‐Related Topics

A self‐devised item measured taken classes on diversity and equity‐related topics: “Have you attended any classes, workshops, or training on diversity and/or social injustice?” Response options were 1 (*yes*) or 0 (*no*). We initially aimed to utilize another follow‐up question assessing the number of hours participants spent in respective classes, with response options being 0 *(no classes*), 1 (*less than 5 h, e.g., one‐time workshop, lecture, etc*.), 2 (*between 5 and 60 h, e.g., a workshop series, several weekend seminars, one to two university courses, etc*.), 3 (*more than 60 h*). Yet, the frequency of response options 2 (*n* = 8) and 3 (*n* = 4) was too low to conduct a robust multiple regression analysis.

#### 7.2.6 Social Dominance Orientation

Participants completed the shortened 8‐item SDO scale (Ho et al. 2015) (e.g., “An ideal society requires that some groups be at the top and others at the bottom”). The response options ranged from 1 (*strongly disagree*) to 8 (*strongly agree*). 4 items were reverse coded, ω = 0.71.

#### 7.2.7 Subjective SES

The German version of the MacArthur Scale of Subjective Socioeconomic Status (Hoebel et al. 2015) assessed participants’ subjective SES. A picture of a ladder was displayed, along with an explanation that this ladder symbolized the composition of German society. Participants were asked: “Where would you place your family of origin (i.e., where you grew up) on the ladder?” Response options ranged from 1 (*lowest*) to 10 (*highest*).

### 7.3 Positionality Statement

The first author is a Turkish woman who took over the project as a doctoral student following the data collection, bringing a critical perspective shaped by her experience as an immigrant in Germany. Her interest in educational disparities and structural barriers is informed by a commitment to challenging meritocratic and deficit‐oriented narratives. The second author is a German‐German woman junior professor who collected the data together with the third author, who is a Turkish postdoctoral researcher, and she has lived and worked in Germany for 7 years. In their research, they explore adolescents’ understanding of cultural diversity and social inequality using interdisciplinary and mixed‐methods approaches. Their deep familiarity with the German education system enriched the interpretation of the findings. Despite differences in cultural and disciplinary backgrounds, all authors share a strong commitment to educational equity and engaged in continuous self‐reflection throughout the research process, particularly during the coding of open‐ended responses.

### 7.4 Coding and Analysis

We analyzed our data qualitatively and descriptively to answer RQ1 and used quantitative methods to address RQ2 and RQ3. All quantitative analyses were conducted in RStudio 2024.04.2. This design corresponds to a data‐transformation variant of a convergent mixed methods study, in which qualitative responses were coded and transformed into quantitative variables that were then integrated into the statistical analysis (Creswell and Plano Clark 2018). The outcome variables for attributions and action intentions were derived from open‐ended responses and analyzed using qualitative content analysis (Mayring 2014). We categorized these responses into relevant attribution or action types using a combination of deductive (theory‐driven) and inductive (data‐driven) methods based on a coding manual. (Syed and Nelson 2015). In the coding manual, open‐ended responses were coded into different subcategories, which formed theoretically meaningful categories. Responses could fall under more than one subcategory. After developing an initial coding manual, two coders who are also preservice teachers were trained by the authors and independently coded the first ten responses for test coding. Consecutively, the coding manual was updated, and the coding process continued with another round. As intercoder reliability was still lower than acceptable (Cohen's Kappa < 0.70), the coding manual was revised again. Although the mean kappa value increased, it still was quite low for some variables (ranging from κ = 0.44 to κ = 1.00, mean κ = 0.80), mainly due to discrepancies emerging from the “other” category (i.e., one coder systematically coded additional attributions into the “other” category, whereas the other coder did not). Whereas Fleiss (1981) considered κ between 0.40 and 0.60 to be fair, Bakeman and Gottman (1986) recommended κ = 0.70 as the lowest acceptable. Considering our high mean kappa value of 0.80 and the substantial number of categories included in our coding manual (Orwin 1994), as well as the fact that the low reliability mainly originated from the “other” category, we chose to proceed with the reliability reported above. Disagreements were resolved through discussion. Of the 93 preservice teachers, one provided no response to the attribution items and nine to the action intentions; these were coded as missing.

Regarding our quantitative analysis, for RQ2, we conducted correlation analyses to examine bivariate relationships among CC subcomponents, as this question aimed to explore associative patterns rather than model explanatory relationships. In contrast, for RQ3, we used multiple regression analyses separately for each outcome variable to identify the unique predictive value of several independent variables while controlling for shared variance. We applied full information maximum likelihood (FIML) to handle missing values (Enders 2022).

Participants’ gender and migration background were included as control variables to account for potential background‐related variation in responses. Prior studies suggest that such characteristics can influence how individuals think about educational inequality (e.g., Glock and Schuchart 2020; Flanagan et al. 2014).

## 8 Results

### 8.1 Qualitative Results

**Figure 2:**
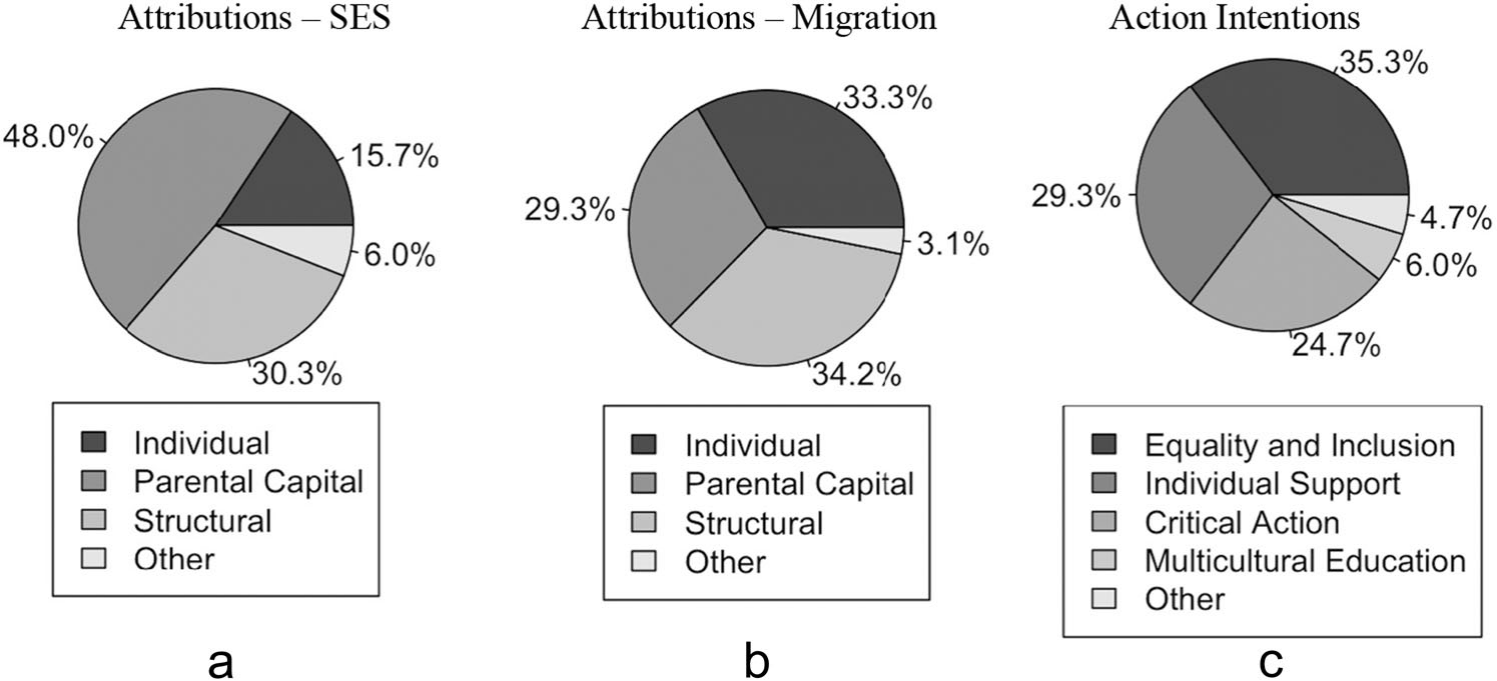
*Frequencies of attributions of educational inequalities and proposed actions to reduce educational inequalities*.

Our qualitative analysis (RQ1) revealed preservice teachers’ attribution and action patterns (Figure 2). Regarding attributions, as expected based on previous research, many participants attributed educational inequality to individual (e.g., effort) and structural (e.g., discrimination) factors (Bañales et al. 2020; Froehlich et al. 2016). In addition to typical individual and structural attributions, many participants attributed educational inequality to parental factors. Some of these were related to parental attitudes towards education. As these placed responsibility on individuals or their culture rather than on the broader system, we merged these with individual attributions (as in Hershberg and Johnson 2019). On the other hand, in our data set, many preservice teachers attributed educational inequalities to gaps in parental educational background, finances, or social capital, which were not clearly individual or structural factors. Therefore, we added parental capital attributions as an additional category to our codebook (Behrmann 2021; Bourdieu 1986). Figure 2 and Tables 1 and 2 show the coding of categories and subcategories for attributions in the SES and migration domains, respectively. Results demonstrate that while participants attributed the achievement gaps between low versus high SES students mainly to parental capital factors (48.0%) followed by structural factors (30.3%), the gap between students with a family history of migration versus those without was mainly attributed to structural factors (34.2%), followed by individual factors (33.3%).

**Table 1:** Coding of categories and subcategories for Preservice Teachers’ attributions of educational inequalities in the SES domain.

Why do students from higher social classes in Germany perform better at school than students from lower social classes?				
Category	Subcategory	Description	Example	Number and % of all SES attributions (*n* = 267)
Individual				15.7% (*n* = 42)
	Prior knowledge	Responses that refer to abilities and skills that the students have learned through special support or upbringing by their parents.	“Because they were often more successfully supported in their education by their environment already in their early childhood…”	3.4% (*n* = 9)
	Effort and motivation	Responses that identify the willingness to make an effort and the learning motivation of the students.	”…students themselves have more ambition”	3.0% (*n* = 8)
	Parental attitudes toward education	Responses that address parents’ attitudes toward school/education/certain learning content/grades and the consequences/expectations of these attitudes.	“Greater interest of parents/families in their children's education”	9.4% (*n* = 25)
Parental Capital				48.0% (*n* = 128)
	Language skills gained in family	Responses that address language or language skills that students have acquired based on their family upbringing/social class‐specific upbringing or through support.	”…understand texts and assignments better because they have a larger vocabulary…”	1.9% (*n* = 5)
	Parental support	Responses that indicate that help/support from parents or the family environment cannot be provided. This help/support pertains to school activities such as doing homework, practicing, or studying.	“Because children from higher social classes receive more support or more is done and paid for them”	16.9% (*n* = 45)
	Parental education	Responses that explicitly mention the educational level of the parents as an explanation.	“The parents often have a better education…”	10.5% (*n* = 28)
	Financial means	Responses that mention the family's financial situation.	“Social inequality usually arises from financial aspects.”	15.4% (*n* = 41)
	Social capital	Responses that cite the social status of the family as well as contacts or networks associated with a specific social class.	“…contact with similar social classes.”	3.4% (*n* = 9)
Structural				30.3% (*n* = 81)
	Access to medical and psychological treatment/exposure to psychological stress	Responses that address systemic issues regarding students’ mental and physical health.	“…due to physical limitations that are not being treated.”	2.6% (*n* = 7)
	Access to educational materials	Responses related to access to educational materials and extra support services.	“Access to tutoring, books, support measures…”	19.9% (*n* = 53)
	Teachers with prejudice	Responses that identify biases or stereotypes of teachers toward different social classes.	“Because teachers grade unevenly. The social background plays a significant role in the assessment of performance.”	4.9% (*n* = 13)
	Societal prejudice	Responses that relate to societal expectations regarding students from certain socioeconomic backgrounds.	“In my opinion, a possible explanation is a certain attitude or expectation of society…”	1.1% (*n* = 3)
	German educational system	Responses that allude to inequalities in the education system.	“There is no real equality of opportunity. The education system is not flexible enough.”	1.9% (*n* = 5)
Other	Additional answers	Responses that cannot be assigned to any of the categories above but still make sense in content.	“…self‐concept.”	6.0% (*n* = 16)

**Table 2:** Coding of categories and subcategories for preservice teachers’ attributions of educational inequalities in the migration domain.

Why do students with a family history of migration perform worse at school than students without?				
Category	Subcategory	Description	Example	Number and % of all migration attributions (*n* = 225)
Individual				33.3% (*n* = 75)
	Language skills	Responses that attribute educational inequalities to students’ lack of language skills.	“Because the lack of German language skills leads to difficulties in all subjects.”	25.8% (*n* = 58)
	Prior knowledge	Responses that attribute educational inequalities to students’ lack of basic knowledge regarding academic content.	“Because they were usually not educated in their home country, resulting in a lack of basic knowledge.”	2.2% (*n* = 5)
	Parental attitudes toward education	Responses that explain educational inequalities as a result of differing priorities or attitudes toward school and education within the family.	“Education is less important to the parents.”	4.0% (*n* = 9)
	Too little efforts to integrate	Responses that place the responsibility for inadequate integration on the individual.	”…Sometimes, they also isolate themselves and spend more time with others from their own background.”	1.3% (*n* = 3)
Parental Capital				29.3% (*n* = 66)
	Parental support	Responses that mention the family support cannot be provided due to parents’ low financial resources and educational level.	“…little support from the parents.”	12% (*n* = 27)
	Parental language skills	Responses that discuss differing language competencies as a possible factor.	“Parents speak a different language at home, so they cannot support their children well”	11.1% (*n* = 25)
	SES	Responses that explicitly refer to the SES (or more specifically financial means) of the family.	“…low SES”	4.4% (*n* = 10)
	Parental education	Responses that specifically refer to the educational level of the parents.	“Educational level of parents”	1.8% (*n* = 4)
Structural				34.2% (*n* = 77)
	Insufficient extra support	Responses that refer to the available or unavailable support opportunities.	“Because they are not supported fairly.”	6.7% (*n* = 15)
	Teachers with prejudice	Responses that refer to discriminatory attitudes of teachers towards pupils.	“I think many teachers are biased against the children.”	10.2% (*n* = 23)
	Peers with prejudice	Responses that are related to the prejudices of peers.	“Exclusion by classmates”	1.8% (*n* = 4)
	Societal prejudice	Responses that reflect societal prejudices.	“Society always associates them with their background and the image they have of that background.”	6.7% (*n* = 15)
	Immigration challenges	Responses that refer to factors originating from the difficulties of immigration.	“Adapting to a new culture and country can cause problems for a child if they have not had to deal with everyday issues, unlike a child without a migration background in the family.”	6.2% (*n* = 14)
	German educational system	Responses that address the factors originating from the structure and organization of the German educational system.	“Disadvantage due to the educational system.”	2.7% (*n* = 6)
Other				
	Additional answers	Responses that cannot be assigned to any of the categories above but still make sense in content.	“Due to a lack of integration or inclusion…”	3.1% (*n* = 7)

Regarding action intentions, preservice teachers mentioned strategies of equality and inclusion, multicultural education, and critical action, in line with previous theory and research (Schachner 2019). Aligning with our definition of critical action in the educational context, critical action strategies encompassed fostering discussions on social inequalities within the school context, as well as intentions to reduce educational inequalities by advocating for systemic change aimed at educational policy and reform. In addition, many preservice teachers proposed engaging in self‐reflection on prejudices and pursuing further education regarding inequalities as potential actions to reduce educational inequalities. As self‐reflection is often viewed as a key factor in praxis for dismantling educational inequalities (Fernández‐Balboa 1998), and as essential to the process of liberation and social justice (Freire^1970^/2018), we included these strategies as a subcategory of critical action in our codebook. In addition, many preservice teachers mentioned employing individualized instruction (Gibbons 1970), and special support for disadvantaged students as strategies to reduce educational inequalities. Therefore, we added “individual support” as an additional category to the codebook. Figure 2 and Table 3 show the coding of categories and subcategories of action intentions. Preservice teachers most frequently mentioned equality and inclusion as a strategy to address educational inequalities (35.3%), followed by individual support (29.3%).

**Table 3:** Coding of categories and subcategories for Preservice teachers’ action intentions to reduce educational inequalities.

What could you do in the future to reduce educational inequalities between students from different groups?				
Category	Subcategory	Description	Example	Number and % of all action intentions (*n* = 150)
Equality and Inclusion				35.3% (*n* = 53)
	Color‐evasion	Responses that neglect cultural/ethnic differences and disadvantages coming from these differences.	“Treating all students equally and promoting them individually.”	2% (*n* = 3)
	Empathy and tolerance	Responses that involve a sensitive and tolerating approach to the issue of educational inequalities.	“Being mindful, being empathetic, being helpful, and providing opportunities.”	6.7% (*n* = 10)
	Reducing prejudice	Responses that identify the unbiased actions and/or judgments of the teacher (also in private life).	“Thinking without prejudice.”	5.3% (*n* = 8)
	Fostering cohesion	Responses that identify the promotion of cohesion among students.	“The sense of community within the class must be strengthened.”	6.7% (*n* = 10)
	Fostering collaboration	Responses that describe encouraging students to support each other or form learning pairs/groups.	“…placing a weaker learner next to a stronger learner …”	4.7% (*n* = 7)
	One‐size‐fits‐all	Responses that include a pedagogical/practical strategy which is expected to fit for every group within classroom.	“Equality, support for students, good education through good teaching.”	2.7% (*n* = 4)
	Inclusion and supporting parents	Responses that outline close collaboration with parents or the active involvement of parents in supporting their child's development.	”…Getting the parents on board”	4.0% (*n* = 6)
	Exchanging with colleagues	Responses that refer to collaborations with colleagues and the school administration.	“…Cooperation with other teachers.”	3.3% (*n* = 5)
Multicultural Education				6.0% (*n* = 9)
	Addressing cultural variations	Responses that describe addressing and/or incorporating various cultures, religions, backgrounds, family situations of students and their families in the classroom.	“Educating the entire group about the various groups so that everyone knows more about each other.”	1.3% (*n* = 2)
	Finding out more about kids’ backgrounds	Responses that mention assessing/asking about the learning conditions of students	“Learning more about the children's backgrounds in the class.”	4.7% (*n* = 7)
Critical Action				24.7% (*n* = 37)
	Self‐reflection	Responses that relate to the self‐reflection of the teacher (also in private life) regarding unbiased actions and/or raising their own awareness of educational inequalities through further training.	“Reflecting on prejudices, etc.”	10.0% (*n* = 15)
	Discussing educational inequalities	Responses that explicitly address educational inequalities (and their reduction) in teaching.	“Raising awareness of educational inequalities…”	10.0% (*n* = 15)
	Engagement with education system and politics	Responses that focus on the involvement of teachers in raising awareness about educational inequalities and/or reducing them both within the school or outside of school (e.g., engaging politically).	“…demanding time and support from the state.”	4.7% (*n* = 7)
Individual Support				29.3% (*n* = 44)
	Differentiated instruction	Responses that relate to the differentiation of instruction for individual students.	“Differentiating my instruction…”	12.7% (*n* = 19)
	Providing special support for language barriers	Responses that address the linguistic barriers.	“Reducing language barriers…”	1.3% (*n* = 2)
	Providing extra support for disadvantaged students	Responses that suggest providing special support for disadvantaged students and for the ones in need.	“Offering support in the form of homework/tutoring”	15.3% (*n* = 23)
Other	Additional	Responses that cannot be assigned to any of the categories above but still make sense in content.	“Strengthening the children's self‐confidence”	6.0% (*n* = 9) 4.7% (*n* = 7)
	No idea	Responses that do not mention any ideas or considerations.	“I don't know that yet.”	1.3% (*n* = 2)

### 8.2 Quantitative Analyses Based on Coded Data

For the outcome variables, we calculated percentage scores (i.e., the percentage of each attribution/action category based on the total number of attributions/actions mentioned by a participant) instead of the sum of the attributions in the respective category to account for variability in the overall number of attributions/actions mentioned. As critical reflection has often been equated to structural attributions in previous research, we used the percentage of structural attributions as an indicator for critical reflection. Similarly, we used the percentage of “critical action” strategies as an outcome variable. We also included the overall number of attributions mentioned by preservice teachers (i.e., quantity of attributions) (see Flanagan et al. 2014). For our regression analyses, we transformed the self‐efficacy variable by dividing each value by 10 due to its high variance because when the ratio of max variance to min variance is greater than 100 among variables, it could present a problem for model iterations.

#### 8.2.1 Hypotheses Testing

Regarding interrelations between CC components (RQ2), bivariate correlations (Table 4) revealed no significant relation between critical action and efficacy (*r* = −0.12, *p* = 0.290) (not supporting H1). Exploratory correlation analyses indicated that there was no significant relation between structural attributions and critical action, nor between structural attributions and efficacy. However, structural attributions in the SES and migration domains were positively related (*r* = 0.27, *p* = 0.008).

**Table 4:** Correlations and descriptives.

	1	2	3	4	5	6	7	8	9	10
1. Discrimination	—									
2. Taken Classes	−0.027	—								
3. SDO	−0.132	0.038	—							
4. Subjective SES	−0.187	0.126	−0.011	—						
5. % Structural Attributions ‐ SES	0.074	0.051	0.004	−0.045	—					
6. % Structural Attributions ‐ Migration	0.165	0.187	0.046	−0.006	0.275**	—				
7. % Critical Action Intentions	0.248*	−0.042	−0.008	−0.215	−0.114	0.075	—			
8. Self‐Efficacy	0.152	0.121	−0.073	0.126	0.082	−0.026	−0.118	—		
9. Gender (0 = male, 1 = female)	0.090	0.021	−0.109	0.044	−0.009	0.133	0.098	0.020	—	
10. Migration Background	0.460**	0.039	−0.106	−0.299**	0.023	0.068	−0.113	0.057	−0.017	—
*M*	1.692	.495	2.161	6.663	0.310	0.325	0.265	44.609	0.879	0.163
SD	0.563	0.503	0.833	1.592	0.258	0.358	0.376	20.190	0.328	0.371

*Note:* Outcome variables were calculated as percentage scores, meaning the proportion of each attribution or action category relative to the total number of responses provided by a participant, to account for variation in how many attributions or actions each participant mentioned in the coded qualitative data.

* *p* < 0.05.

** *p* < 0.01.

Regarding predictors (RQ3), multiple regression analyses (Table 5) revealed that discrimination experiences positively predicted critical action intentions (*b* = 0.22, *SE* = 0.08, *p* = 0.007) (partially supporting H2a). Having taken classes on diversity and equity‐related topics did not predict structural attributions in the SES (*b* = 0.03, *SE* = 0.05, *p* = 0.562) or migration domain (*b* = 0.00, *SE* = 0.02, *p* = 0.849), nor did it predict self‐efficacy (*b* = 0.46, *SE* = 0.41, *p* = 0.267) (not supporting H2b). However, having taken classes on diversity and equity‐related topics positively (*b* = 0.49, *SE* = 0.22, *p* = 0.029) predicted the number of attributions that were given in the SES domain (supporting H2b). SDO did not significantly predict either structural attributions in the SES (*b* = 0.00, *SE* = 0.03, *p* = 0.957) or migration domain (*b* = 0.03, *SE* = 0.04, *p* = 0.466) nor critical action (*b* = 0.01, *SE* = 0.05, *p* = 0.821) or self‐efficacy (*b* = ‐0.10, *SE* = 0.25, *p* = 0.676) (not supporting H2c). We explored the relation between subjective SES and CC subcomponents. Subjective SES negatively predicted critical action intentions (*b* = 0.22, *SE* = 0.08, *p* = 0.007).

**Table 5:** Results from multiple regression analyses.

	% Structural Attributions ‐ SES					% Structural Attributions ‐ Migration					% Critical Action Intentions					Self‐Efficacy				
			95% CI					95% CI					95% CI					95% CI		
	*b*	*SE*	*LL*	*UL*	*p*	*b*	*SE*	*LL*	*UL*	*p*	*b*	*SE*	*LL*	*UL*	*p*	*b*	*SE*	*LL*	*UL*	*p*
**Predictors**																				
Discrimination	0.04	0.05	−0.07	0.14	0.484	0.11	0.07	−0.03	0.25	0.139	0.22	0.08	0.06	0.37	0.007	0.65	0.42	−0.17	1.47	0.122
Taken Classes	0.03	0.05	−0.07	0.14	.562	0.00	0.02	−0.03	0.04	0.849	0.01	0.08	−0.14	0.16	0.917	0.46	0.41	−0.35	1.26	0.267
SDO	0.00	0.03	−0.06	0.07	0.957	0.03	0.04	−0.05	0.12	0.466	0.01	0.05	−0.08	0.10	0.821	−0.10	0.25	−0.60	0.39	0.676
Subjective SES	−0.01	0.02	−0.04	0.03	0.683	−0.00	0.02	−0.05	0.05	0.983	−0.06	0.02	−0.11	−0.01	0.013	0.19	0.14	−0.08	0.46	0.170
**Control Variables**																				
Gender (0 = male, 1 = female)	−0.02	0.09	−0.19	0.15	0.856	0.12	0.12	−0.11	0.36	0.292	0.15	0.14	−0.12	0.42	0.287	−0.13	0.66	−1.43	1.18	0.850
Migration Background	−0.02	0.08	−0.07	0.15	0.824	−0.00	0.11	−0.23	0.22	0.998	−0.33	0.12	−0.57	−0.10	0.005	0.09	0.64	−1.18	1.35	0.133

*Note:* Outcome variables were calculated as percentage scores, meaning the proportion of each attribution or action category relative to the total number of responses provided by a participant, to account for variation in how many attributions or actions each participant mentioned in the coded qualitative data.

We also conducted exploratory analyses on additional types of attributions and action intentions not covered by our main hypotheses. The results of these analyses and their correlations are presented in the Supporting Material (e.g., Tables 7,8, and 9).

#### 8.2.2 Robustness Check

We additionally conducted robustness checks without control variables to examine the stability of our findings. Table 6 (see Supporting Material) displays the multiple regression analyses. While discrimination experiences still significantly predicted critical action intentions (*b* = 0.17, *SE* = 0.08, *p* = 0.032), the effect of subjective SES on critical action intentions was not significant anymore (*b* = −0.04, *SE* = 0.02, *p* = 0.109).

## 9 Discussion

In light of persistent educational inequalities, it is crucial that (preservice) teachers develop CC. This includes reflecting on structural causes (critical reflection), feeling empowered to address them (self‐efficacy), and engaging in change‐oriented action (critical action). This mixed‐methods study explored German preservice teachers’ attributions and action intentions regarding educational inequalities, examining the interrelations between CC subcomponents and their predictors.

### 9.1 RQ1: Preservice Teachers’ Attributions and Actions in the Context of Educational Inequalities

Structural attributions made up 30.3% of responses in the SES domain, with most focusing on access to educational resources such as textbooks and digital tools (19.9%). Less attention was given to broader systemic factors like healthcare access or institutional bias, indicating limited awareness of deeper structural issues. Nearly half of the SES‐related attributions (48.0%) referred to parental capital. Since students lack their own economic and educational resources, their circumstances are largely shaped by their parents. Many preservice teachers highlighted forms of capital resembling Bourdieu's concept of economic, social, and cultural capital (1986), a widely influential framework in German education (Müller 2021). The most frequently mentioned aspect was parents’ ability to support their children, consistent with Behrmann (2021) findings.

Some attributions reflected deficit‐oriented views, blaming parents for not valuing education. Following Hershberg and Johnson (2019), we coded these as individual attributions, which formed the largest share in this category. Preservice teachers thus explained both SES‐ and migration‐related inequalities through parental values, engaging in “culture of poverty” thinking that may reinforce stereotypes.

Structural attributions were most common for migration‐related inequalities (34.2%), aligning with prior research in Germany (Glock and Schuchart 2020). Teacher prejudice was the most frequently mentioned factor among structural attributions, likely due to its impact on grading and tracking decisions that shape students’ academic paths (Sprietsma 2013; Forster 2021). This may explain why preservice teachers see it as a key contributor to opportunity gaps.

Individual attributions for migration‐related inequalities remained high (33.3%), mostly focusing on students’ German language skills. Given Germany's strong monolingual norms and the importance of subject‐specific language, language proficiency is closely tied to academic success (Lehmann et al. 1995; Gogolin et al. 2019). However, the system still inadequately supports language learners, often placing them into lower school tracks before they can develop sufficient skills, thus reinforcing inequalities (Güllüpınar and Fernández‐Kelly 2012).

Drawing on prior research on cultural racism in German education (Moffitt et al. 2019), we expected some preservice teachers to explain migration‐related inequalities through deficit‐oriented cultural attributions. While a few linked these inequalities to parental educational values, such attributions were rare (5.3%). This contrasts with findings showing that students with a family history of migration often place a higher value on education than those without (Hadjar and Scharf 2019). While some preservice teachers attributed migration‐related inequalities to parents not valuing education, more (29.3%) referred to family‐related factors like income, support, and education. Still, parental capital was mentioned less often for migration‐related inequalities than for SES‐related ones, despite migration also being associated with a loss of resources and capital.

Overall, this mixed‐methods study shows that preservice teachers’ explanations for SES‐ and migration‐related inequalities go beyond a simple individual‐versus‐structural divide. Family and cultural factors, such as the value placed on education, also shape their attributions. Notably, teachers’ instructional quality was rarely considered, despite its well‐documented influence on student success (Hattie 2003).

In terms of action intentions, “equality and inclusion” was the most common strategy preservice teachers suggested to address educational inequalities (35.3%), reflecting its strong emphasis in German classroom diversity climates (Civitillo et al. 2017). Many preservice teachers focused their proposed actions on promoting empathy, tolerance, cohesion, and contact—concepts that, although important for reducing prejudice, may remain insufficient for driving meaningful change without addressing underlying power disparities (Tropp et al. 2021).

“Individual support” (29.3%) was the second most common action strategy, mainly involving differentiated instruction and special support for disadvantaged students (Gibbons 1970). Though unexpected from our initial review, it appears easier and less controversial for teacher candidates to adjust teaching methods than to challenge the broader educational system.

We classified 24.7% of action intentions as “critical actions,” mainly involving self‐reflection and classroom discussions on educational inequalities. Although self‐reflection may not traditionally be considered critical action (Mathews et al. 2023), we view it as a vital first step in challenging existing biases and social positions (Fernández‐Balboa 1998). The emphasis on discussing inequalities aligns with Freire (1970/2018) concept of problem‐posing education, where teachers and students collaboratively address social issues. Only a few preservice teachers suggested actions targeting systemic change, likely because German teacher education primarily focuses on equality and inclusion (Reis et al. 2020) and individualized instructional strategies (Kultusministerkonferenz KMK 2016), making classroom‐level actions seem more achievable than broader structural reforms.

Meanwhile, “multicultural education” was the least mentioned action strategy among preservice teachers (6.0%). This reflects the German context, which still favors an assimilative societal climate over multiculturalism (Civitillo et al. 2017). As a result, the focus remains on equality and inclusion rather than multicultural education or critical consciousness socialization in the classroom (Schachner et al. 2021).

### 9.2 RQ2: Interrelations between CC Components

We examined the relationships between CC subcomponents. Contrary to our hypothesis (H1), no link was found between critical action and self‐efficacy. Our definition of critical action is specific to the educational context, which may differ from traditional definitions (e.g., Watts et al. 2011). Additionally, preservice teachers might feel limited in taking action due to external pressures like school principals or heavy workloads, which can hinder their intentions despite motivation and readiness.

We also explored the relationship between structural attributions and critical actions but found no correlation. As Chubbuck (2010) notes, the complex nature of attributions does not always lead to a single approach to addressing opportunity gaps, which may explain our findings. However, our exploratory analysis revealed a positive link between structural attributions in the SES and migration domains, consistent with Diemer et al. (2017). This suggests that adopting an equity perspective supports a critical understanding of inequalities across different areas, especially considering the intersection of SES and migration issues (SVR‐Forschungsbereich 2016).

### 9.3 RQ3: Predictors of CC

Discrimination was examined as a potential predictor of structural attributions and critical action but was only positively linked to critical action, partially supporting our hypothesis (H2a). The absence of a connection with structural attributions may indicate that preservice teachers who have not reached a certain level of reflection might struggle to recognize discrimination as a systemic issue. Additionally, discrimination experiences may lead them to prioritize structural attributions less, potentially as a way to maintain hope for succeeding in life (Godfrey and Wolf 2016).

Contrary to our expectation (H2b), taking classes on diversity and equity‐related topics in the past did not predict structural attributions or self‐efficacy to address educational inequality, despite prior research suggesting such links (Rapa et al. 2022). However, prior course participation was associated with a higher number of attributions in the SES (but not migration) domain. Since attributional complexity is often tied to deeper reflection (Flanagan et al. 2014), this may indicate that learning about inequalities helps preservice teachers recognize multiple contributing factors. Still, most participants reported fewer than 5 h of relevant training, usually through one‐off workshops or lectures. This limited exposure likely reflects both curricular gaps in teacher education and the sociocultural context of the university, located in a relatively homogenous area of East Germany.

SDO was not significantly associated with any CC subcomponents, thus not supporting H2c. One explanation may be that an unjust system benefits privileged groups like preservice teachers. While they might recognize inequality to some extent, this awareness may not lead to action, as it could conflict with their own interests in maintaining the status quo. Moreover, SDO captures a broader ideological orientation toward group‐based hierarchy, whereas our operationalization of critical reflection focused more narrowly on structural attributions for educational inequality. This conceptual mismatch may also explain the absence of a link.

Subjective SES negatively predicted critical action intentions in our analysis, indicating that preservice teachers with higher SES were less likely to engage in critical action, consistent with CC theory. Preservice teachers with low SES may identify with and empathize with students in similar circumstances, potentially encouraging them to engage in critical actions toward a more equitable system.

## 10 Strengths, Limitations and Further Directions

A key strength of our study is the mixed‐methods approach, combining open‐ended questions with rating‐scale items to investigate CC and its antecedents. This approach, recommended for capturing a nuanced understanding of CC (Godfrey and Burson 2018), allowed us to explore the full range of teachers’ attributions and actions. Consequently, we identified additional types of attributions (e.g., parental capital) and actions (e.g., individualized instruction) that previous research had not included. By including all three CC subcomponents, we could explore their interrelations and integrate insights from distinct research strands such as attributions, diversity climate, and self‐efficacy. Unlike most CC studies centered in the US, our focus on preservice teachers in Germany adds to a less explored context. Importantly, we highlight the role of CC among privileged future teachers, an often neglected yet critical group for social justice (Chubbuck 2010; Jemal 2017; Thomas et al. 2014).

One limitation of the study is its cross‐sectional design, which prevents examination of changes over time. A longitudinal approach or inclusion of the second measurement point (excluded due to high dropout) could have offered deeper insights into CC development. While the sample size was sufficient for detecting medium effects, it was limited for identifying smaller ones. A larger sample may have yielded more significant results, particularly for variables that showed marginal significance in our current analyses.

The sample, drawn from East Germany (a region with low ethnic and cultural diversity), may not reflect patterns found in more diverse contexts like West Germany. Additionally, although SES and migration history are closely linked in Germany (SVR‐Forschungsbereich 2016), we assessed them separately, making it unclear whether participants considered their intersection. Future studies could use vignettes to examine intersecting identities, including factors such as religion or gender.

Another limitation of the present study concerns the framing of the open‐ended attribution questions. Specifically, the question regarding SES was framed in terms of relative advantage, and the question concerning migration background in terms of relative disadvantage. They may have unintentionally influenced participants’ attribution styles, potentially eliciting more structural explanations in one domain and more individual or cultural explanations in the other.

## 11 Implications and Conclusion

Our findings suggest that in regions like East Germany, where diversity among preservice teachers is limited, teacher education should more explicitly address educational inequalities and institutional discrimination. Programs could deepen understanding of systemic factors such as stereotyping, tracking, and the compounding effects of low SES and migration histories, especially in contexts with limited exposure to diversity. While emphasizing equality and inclusion is important, this focus can risk promoting color‐evasion and assimilation. Teacher education should therefore foster both critical reflection and a sense of agency, hope, and action.

In Germany, legal frameworks such as the Neutrality Law and the Civil Servant Act restrict partisan expressions or strikes but still require teachers to uphold equal opportunity and democratic values. Addressing discrimination or right‐wing extremism aligns with this responsibility, as long as it avoids indoctrination (Wieland 2019).

Further research is needed to understand the antecedents of CC among preservice teachers, which could inform the design of targeted interventions and teacher training programs aimed at fostering social justice education. By identifying key factors influencing CC, these programs can equip preservice teachers with the tools and knowledge to address educational inequalities effectively in their future classrooms.

## Ethics Statement

This study was approved by the University of Potsdam Ethics Committee and was conducted in accordance with the principles outlined in the Declaration of Helsinki.

## Consent

Informed consent was obtained from all participants included in the study.

## Conflicts of Interest

The authors declare no conflicts of interest.

## Permission to Reproduce Material From Other Sources

Not applicable.

## Referee Suggestions

1. Josefina Bañales, jbanal2@uic.edu, University of Illinois Chicago. Expertise in critical consciousness, antiracism, civic engagement, and sociopolitical development.
2. Laura Froehlich, laura.froehlich@fernuni-hagen.de, FernUniversität in Hagen. Expertise in stereotypes about ethnicity and gender, social identity, and integration of immigrants in education.
3. Jolina Ulbricht, jolina.ulbricht@geo.uni-halle. de, Martin‐Luther‐Universität Halle‐Wittenberg. Expertise in intercultural self‐efficacy and cultural beliefs of teachers.

## Supporting information

Supplementary Tables.

## Data Availability

Data is not publicly available, as the participant consent form did not include a request for permission to release the data. However, all study materials and syntax are available at https://osf.io/y846a.
