# Supplementary material for: How Do Preservice Teachers Make Sense of Educational Inequalities? Exploring Critical Consciousness Through Mixed Methods
Source: J Community Psychol. 2025 Dec 2;54(1):e70070. doi: 10.1002/jcop.70070 (PMC12671997; doi:10.1002/jcop.70070)
Supplement: Supplementary file 1 — Supplementary Tables. [file JCOP-54-0-s001.docx]

**Supplementary Materials**

***Additional Analyses***

Although we did not set specific hypotheses about other types of attributions (e.g., individual attributions, parental capital attributions) and action intentions (e.g., equality and inclusion, multicultural education, and individualized support), we explored their relations with our predictors, using separate multiple regressions for each outcome (Tables 7 and 8). Subjective SES positively predicted parental capital attributions in the SES domain (*b* = 0.04, *SE* = 0.02, *p* = .023). Female gender predicted fewer individual attributions in the migration domain (*b* = -0.25, *SE* = 0.11, *p* = .030). Higher levels of subjective SES predicted more equality and inclusion action intentions (*b* = 0.05, *SE* = 0.02, *p* = .036). See Table 9 for correlations and descriptives among all variables.

**Table 6**

*Results from Multiple Regression Analysis Without Control Variables*

|  | **Structural Attributions - SES** | | | | | **Structural Attributions - Migration** | | | | | **Critical Action Intentions** | | | | | **Self-Efficacy** | | | | |
| --- | --- | --- | --- | --- | --- | --- | --- | --- | --- | --- | --- | --- | --- | --- | --- | --- | --- | --- | --- | --- |
|  | *b* | *SE* | 95% CI | | *p* | *b* | *SE* | 95% CI | | *p* | *b* | *SE* | 95% CI | | *p* | *b* | *SE* | 95% CI | | *p* |
|  |  |  | *LL* | *UL* |  |  |  | *LL* | *UL* |  |  |  | *LL* | *UL* |  |  |  | *LL* | *UL* |  |
| **Predictors** | | | | | | | | | | | | | | | | | | | | |
| Discrimination | 0.03 | 0.05 | -0.06 | 0.13 | .512 | 0.11 | 0.06 | -0.01 | 0.24 | .083 | 0.17 | 0.08 | 0.01 | 0.31 | .032 | 0.66 | 0.38 | -0.09 | 1.41 | .085 |
| Taken Classes | 0.03 | 0.05 | -0.08 | 0.13 | .584 | 0.13 | 0.07 | -0.01 | 0.28 | .063 | -0.02 | 0.08 | -0.17 | 0.14 | .815 | 0.45 | 0.41 | -0.34 | 1.27 | .265 |
| SDO | 0.00 | 0.03 | -0.06 | 0.07 | .926 | 0.03 | 0.04 | -0.06 | 0.11 | .550 | 0.03 | 0.05 | -0.07 | 0.12 | .569 | -0.10 | 0.25 | -0.59 | 0.38 | .067 |
| Subjective SES | -0.01 | 0.02 | -0.04 | 0.03 | .716 | 0.00 | 0.02 | -0.04 | 0.05 | .973 | -0.04 | 0.02 | -0.09 | 0.01 | .109 | 0.19 | 0.13 | -0.07 | 0.45 | .154 |

**Table 7**

*Multiple Regression Analysis with Attribution Categories*

|  | **Structural - SES** | | | | | **Individual - SES** | | | | | **Parental Capital - SES** | | | | | | **Structural - Migration** | | | | | **Individual - Migration** | | | | | **Parental - Migration** | | | | |
| --- | --- | --- | --- | --- | --- | --- | --- | --- | --- | --- | --- | --- | --- | --- | --- | --- | --- | --- | --- | --- | --- | --- | --- | --- | --- | --- | --- | --- | --- | --- | --- |
|  | *b* | *SE* | 95% CI | | *p* | *b* | *SE* | 95% CI | | *p* | *b* | *SE* | 95% CI | | *p* | *b* | | *SE* | 95% CI | | *p* | *b* | *SE* | 95% CI | | *p* | *b* | *SE* | 95% CI | | *p* |
|  |  |  | *LL* | *UL* |  |  |  | *LL* | *UL* |  |  |  | *LL* | *UL* |  |  | |  | *LL* | *UL* |  |  |  | *LL* | *UL* |  |  |  | *LL* | *UL* |  |
| **Predictors** | | | | | | | | | | | | | | | | | | | | | |  | | | | |  | | | | |
| Discrimination | 0.04 | 0.05 | -0.07 | 0.14 | .484 | -0.03 | 0.05 | -0.13 | 0.07 | .545 | -0.02 | 0.06 | -0.14 | 0.09 | .711 | 0.11 | | 0.07 | -0.03 | 0.25 | .139 | -0.09 | 0.07 | -0.22 | 0.05 | .227 | 0.00 | 0.06 | -0.12 | 0.13 | .937 |
| Taken Classes | 0.03 | 0.05 | -0.07 | 0.14 | .562 | -0.01 | 0.05 | -0.11 | 0.09 | .851 | 0.01 | 0.06 | -0.10 | 0.13 | .816 | 0.00 | | 0.02 | -0.03 | 0.04 | .849 | -0.15 | 0.07 | -0.29 | -0.01 | .038 | 0.01 | 0.06 | -0.12 | 0.14 | .889 |
| SDO | 0.00 | 0.03 | -0.06 | 0.07 | .957 | -0.00 | 0.03 | -0.06 | 0.06 | .904 | -0.03 | 0.04 | -0.10 | 0.04 | .362 | 0.03 | | 0.04 | -0.05 | 0.12 | .466 | 0.04 | 0.04 | -0.04 | 0.13 | .337 | -0.05 | 0.04 | -0.12 | 0.03 | .234 |
| Subjective SES | -0.01 | 0.02 | -0.04 | 0.03 | .683 | -0.03 | 0.02 | -0.06 | 0.00 | .065 | 0.04 | 0.02 | 0.01 | 0.08 | .023 | -0.00 | | 0.02 | -0.05 | 0.05 | .983 | 0.02 | 0.02 | -0.02 | 0.07 | .330 | -0.02 | 0.02 | -0.06 | 0.02 | .298 |
| **Control Variables** | | | | | | | | | | | | | | | | | | | | | |  | | | | |  | | | | |
| Gender (0 = male, 1 = female) | -0.02 | 0.09 | -0.19 | 0.15 | .856 | 0.12 | 0.08 | -0.04 | 0.28 | .144 | -0.15 | 0.09 | -0.33 | 0.04 | .115 | 0.12 | | 0.12 | -0.11 | 0.36 | .292 | -0.25 | 0.11 | -0.47 | -0.02 | .030 | 0.15 | 0.10 | -0.05 | 0.35 | .144 |
| Migration Background | -0.02 | 0.08 | -0.07 | 0.15 | .824 | 0.05 | 0.08 | -0.10 | 0.21 | .512 | 0.01 | 0.09 | -0.17 | 0.19 | .923 | -0.00 | | 0.11 | -0.23 | 0.22 | .998 | 0.02 | 0.11 | -0.20 | 0.24 | .863 | -0.10 | 0.10 | -0.29 | 0.10 | .336 |
|  |  |  |  |  |  |  |  |  |  |  |  |  |  |  |  |  | |  |  |  |  |  |  |  |  |  |  |  |  |  |  |

**Table 8**

*Multiple Regression Analysis with Action Intention Categories*

|  | **Equality and Inclusion** | | | | | **Multicultural Education** | | | | | **Critical Action** | | | | | **Individual Support** | | | | |
| --- | --- | --- | --- | --- | --- | --- | --- | --- | --- | --- | --- | --- | --- | --- | --- | --- | --- | --- | --- | --- |
|  | *b* | *SE* | 95% CI | | *p* | *b* | *SE* | 95% CI | | *p* | *b* | *SE* | 95% CI | | *p* | *b* | *SE* | 95% CI | | *p* |
|  |  |  | *LL* | *UL* |  |  |  | *LL* | *UL* |  |  |  | *LL* | *UL* |  |  |  | *LL* | *UL* |  |
| **Predictors** | | | | | | | | | | | | | | | | | | | | |
| Discrimination | -0.13 | 0.08 | -0.28 | 0.02 | .100 | -0.04 | 0.04 | -0.11 | 0.04 | .318 | 0.22 | 0.08 | 0.06 | 0.37 | .007 | 0.00 | 0.08 | -0.16 | 0.16 | .974 |
| Taken Classes | -0.07 | 0.07 | -0.21 | 0.08 | .374 | -0.04 | 0.04 | -0.11 | 0.03 | .264 | 0.01 | 0.08 | -0.14 | 0.16 | .917 | -0.03 | 0.08 | -0.18 | 0.12 | .691 |
| SDO | -0.04 | 0.05 | -0.13 | 0.05 | .416 | -0.02 | 0.02 | -0.06 | 0.03 | .400 | 0.01 | 0.05 | -0.08 | 0.10 | .821 | 0.00 | 0.05 | -0.09 | 0.10 | .911 |
| Subjective SES | 0.05 | 0.02 | 0.00 | 0.10 | .036 | 0.00 | 0.01 | -0.02 | 0.02 | .996 | -0.06 | 0.02 | -0.11 | -0.01 | .013 | 0.02 | 0.02 | -0.03 | 0.06 | .540 |
| **Control Variables** | | | | | | | | | | | | | | | | | | | | |
| Gender (0 = male, 1 = female) | 0.04 | 0.13 | -0.22 | 0.30 | .774 | -0.01 | 0.06 | -0.13 | 0.12 | .931 | 0.15 | 0.14 | -0.12 | 0.42 | .287 | -0.24 | 0.14 | -0.51 | 0.03 | .088 |
| Migration Background | 0.17 | 0.12 | -0.05 | 0.40 | .131 | 0.00 | 0.06 | -0.11 | 0.11 | .976 | -0.33 | 0.12 | -0.57 | -0.10 | .005 | 0.00 | 0.12 | -0.23 | 0.24 | .973 |

**Table 9**

*Correlations and Descriptives*

|  | **1.** | **2.** | **3.** | **4.** | **5.** | **6.** | **7.** | **8.** | **9.** | **10.** | **11.** | **12.** | **13.** | **14.** | **15.** | **16.** | **17.** | **18.** | **19.** | **20.** |
| --- | --- | --- | --- | --- | --- | --- | --- | --- | --- | --- | --- | --- | --- | --- | --- | --- | --- | --- | --- | --- |
| **Predictors** |  |  |  |  |  |  |  |  |  |  |  |  |  |  |  |  |  |  |  |  |
| 1. Discrimination | – |  |  |  |  |  |  |  |  |  |  |  |  |  |  |  |  |  |  |  |
| 2. Taken Classes | -.027 | – |  |  |  |  |  |  |  |  |  |  |  |  |  |  |  |  |  |  |
| 3. SDO | -.132 | .038 | – |  |  |  |  |  |  |  |  |  |  |  |  |  |  |  |  |  |
| 4. Subjective SES | -.187 | .126 | -.011 | – |  |  |  |  |  |  |  |  |  |  |  |  |  |  |  |  |
| **Attributions %** |  |  |  |  |  |  |  |  |  |  |  |  |  |  |  |  |  |  |  |  |
| 5. Structural - SES | .074 | .051 | .004 | -.045 | – |  |  |  |  |  |  |  |  |  |  |  |  |  |  |  |
| 6. Individual - SES | .019 | -.033 | -.031 | -.201 | -.379^**^ | – |  |  |  |  |  |  |  |  |  |  |  |  |  |  |
| 7. Parental Capital - SES | -.085 | .045 | -.069 | .241^*^ | -.485^**^ | -.426^**^ | – |  |  |  |  |  |  |  |  |  |  |  |  |  |
| 8. SES - Quantity | -.168 | .240^*^ | .080 | .035 | -.063 | -.003 | .082 | – |  |  |  |  |  |  |  |  |  |  |  |  |
| 9. Structural - Migration | .165 | .187 | .046 | -.006 | .275^**^ | -.347^**^ | -.047 | .162 | – |  |  |  |  |  |  |  |  |  |  |  |
| 10. Individual - Migration | -.172 | -.195 | .130 | .086 | -.220^*^ | .219^*^ | .013 | -.280^**^ | -.605^**^ | – |  |  |  |  |  |  |  |  |  |  |
| 11. Parental Capital - Migration | .011 | -.004 | -.131 | -.072 | -.051 | .136 | .014 | .138 | -.385^**^ | -.445^**^ | – |  |  |  |  |  |  |  |  |  |
| 12. Migration – Quantity  **Actions %** | -.159 | -.052 | .006 | .114 | .024 | -.105 | .120 | .388^**^ | .087 | -.323^**^ | .278^**^ | – |  |  |  |  |  |  |  |  |
| 13. Critical Action | .248^*^ | -.042 | -.008 | -.215 | -.114 | .184 | -.090 | -.158 | .075 | -.015 | -.086 | -.036 | – |  |  |  |  |  |  |  |
| 14. Equality and Inclusion | -.134 | -.057 | -.079 | .207 | -.009 | -.049 | .013 | .019 | .094 | .102 | -.213 | -.043 | -.430^**^ | – |  |  |  |  |  |  |
| 15. Multicultural Education | -.096 | -.130 | -.069 | .001 | -.090 | -.055 | .116 | .037 | -.066 | .068 | .029 | .136 | -.192 | -.039 | – |  |  |  |  |  |
| 16. Individual Support | -.050 | -.041 | .027 | .043 | .077 | -.011 | .019 | .050 | -.201 | -.018 | .264^*^ | .088 | -.399^**^ | -.351^**^ | -.163 | – |  |  |  |  |
| 17. Actions - Quantity | -.122 | .025 | -.113 | .135 | -.063 | -.041 | .122 | .189 | .098 | -.072 | -.026 | .366^**^ | -.100 | .210 | .087 | .010 | – |  |  |  |
| 18. Critical Motivation  **Control Variables** | .152 | .121 | -.073 | .126 | .082 | .001 | -.041 | .227^*^ | -.026 | -.045 | .070 | .035 | -.118 | -.034 | .120 | .094 | -.046 | – |  |  |
| 19. Gender (0 = male, 1 = female) | .090 | .021 | -.109 | .044 | -.009 | .131 | -.145 | .000 | .133 | -.255^*^ | .163 | .211^*^ | .098 | .035 | -.032 | -.182 | .102 | .020 | – |  |
| 20. Migration Background | .460^**^ | .039 | -.106 | -.299^**^ | .023 | .102 | -.059 | .070 | .068 | -.079 | -.065 | -.133 | -.113 | .065 | -.030 | -.046 | -.147 | .057 | -.017 | – |
| *M* | 1.692 | .495 | 2.161 | 6.663 | .310 | .158 | .470 | 2.902 | .325 | .398 | .245 | 2.419 | .265 | .317 | .053 | .292 | 1.786 | 44.609 | .879 | .163 |
| *SD* | .563 | .503 | .833 | 1.592 | .258 | .246 | .294 | 1.130 | .358 | .364 | .315 | 1.370 | .376 | .350 | .167 | .353 | .945 | 20.190 | .328 | .371 |

*Note.* *p<.05. **p<.01.
